# Comparative cell-specific transcriptomics reveals differentiation of C_4_ photosynthesis pathways in switchgrass and other C_4_ lineages

**DOI:** 10.1093/jxb/erv553

**Published:** 2016-02-19

**Authors:** Xiaolan Rao, Nan Lu, Guifen Li, Jin Nakashima, Yuhong Tang, Richard A. Dixon

**Affiliations:** ^1^Department of Biological Sciences, University of North Texas, 1155 Union Circle #305220, Denton, TX 76203, USA; ^2^BioEnergy Science Center (BESC), US Department of Energy, Oak Ridge, TN 37831, USA; ^3^Samuel Roberts Noble Foundation, 2510 Sam Noble Parkway, Ardmore, OK 73401, USA

**Keywords:** C_4_ photosynthesis, carbon fixation, cell-specific transcriptomics, comparative transcriptomics, switchgrass.

## Abstract

Almost all C_4_ plants require the co-ordination of the adjacent and fully differentiated cell types, mesophyll (M) and bundle sheath (BS). The C_4_ photosynthetic pathway operates through two distinct subtypes based on how malate is decarboxylated in BS cells; through NAD-malic enzyme (NAD-ME) or NADP-malic enzyme (NADP-ME). The diverse or unique cell-specific molecular features of M and BS cells from separate C_4_ subtypes of independent lineages remain to be determined. We here provide an M/BS cell type-specific transcriptome data set from the monocot NAD-ME subtype switchgrass (*Panicum virgatum*). A comparative transcriptomics approach was then applied to compare the M/BS mRNA profiles of switchgrass, monocot NADP-ME subtype C_4_ plants maize and *Setaria viridis*, and dicot NAD-ME subtype *Cleome gynandra*. We evaluated the convergence in the transcript abundance of core components in C_4_ photosynthesis and transcription factors to establish Kranz anatomy, as well as gene distribution of biological functions, in these four independent C_4_ lineages. We also estimated the divergence between NAD-ME and NADP-ME subtypes of C_4_ photosynthesis in the two cell types within C_4_ species, including differences in genes encoding decarboxylating enzymes, aminotransferases, and metabolite transporters, and differences in the cell-specific functional enrichment of RNA regulation and protein biogenesis/homeostasis. We suggest that C_4_ plants of independent lineages in both monocots and dicots underwent convergent evolution to establish C_4_ photosynthesis, while distinct C_4_ subtypes also underwent divergent processes for the optimization of M and BS cell co-ordination. The comprehensive data sets in our study provide a basis for further research on evolution of C_4_ species.

## Highlight

Determination of mesophyll and bundle sheath cell-specific transcriptomes for the monocot NAD-ME C_4_ plant switchgrass reveals both evolutionary divergence and common elements in C_4_ establishment.

## Introduction

C_4_ species are among the world’s most important food, feed, and bioenergy crops, including maize (*Zea mays*), sugarcane (*Saccharum officinarum*), sorghum (*Sorghum bicolor*), and switchgrass (*Panicum virgatum*) (Brutnell *et al.*, 2010; John *et al.*, 2014). C_4_ plants have evolved the C_4_ cycle pathway, which creates a CO_2_ pump that concentrates CO_2_ around the carboxylating enzyme Rubisco (Christin *et al.*, 2009). In most C_4_ species, this is achieved through integrating the two CO_2_ assimilation pathways spatially into two discrete cell types, namely mesophyll (M) and bundle sheath (BS) cells (Sheen, 1999; John *et al.*, 2014). C_4_ plants can achieve high photosynthetic efficiency and consequently decrease photorespiration. These characteristics enable C_4_ plants to thrive in tropical and subtropical environments that induce high rates of photorespiration in C_3_ plants (Brutnell *et al.*, 2010).

**Fig. 1.**
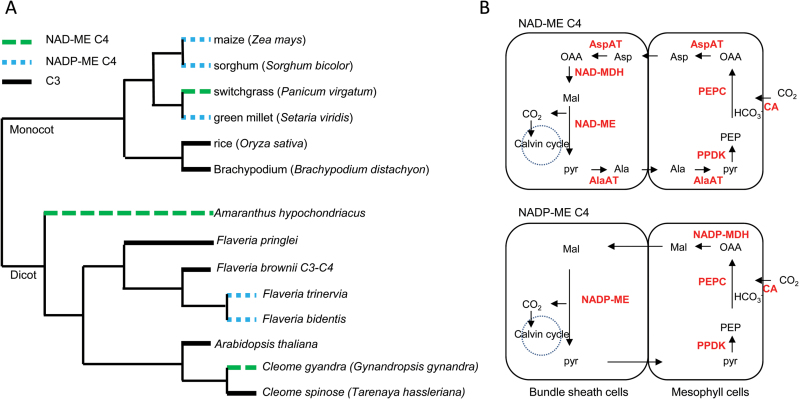
Phylogenetic tree of selected C_4_ and C_3_ species and carbon fixation process of C_4_ leaves. (A) Phylogenetic analysis of C_4_ model species with rice, Arabidopsis, and Brachypodium C_3_ model species, drawn based on Bennetzen *et al.* (2012). (B) The major biochemical cycles in NAD-ME and NADP-ME subtypes of C4 photosynthesis. AlaAT, aspartate aminotransferase; AspAT, aspartate aminotransferase; CA, carbonic anhydrase; PEPC, phosphoenolpyruvate carboxylase; PEPCK, phosphoenolpyruvate carboxykinase; NAD-ME, NAD-dependent malic enzyme; NAD-MDH, NAD-dependent malate dehydrogenase; NADP-ME, NADP-dependent malic enzyme; NADP-MDH, NADP-dependent malate dehydrogenase; PPDK, pyruvate/orthophosphate dikinase; Ala, alanine; Asp, aspartate; Mal, malate; Pyr, pyruvate; OAA, oxaloacetic acid; PEP, phosphoenolpyruvate. (This figure is available in colour at *JXB* online.)

In C_4_ plants, CO_2_ fixation is a two-step process. Atmospheric CO_2_ is initially fixed by phosphoenolpyruvate carboxylase (PEPC) in the cytosol of M cells. The resulting four-carbon dicarboxylic acid oxaloacetate (OAA) is converted to malate or aspartate. These C_4_ acids are then transferred into the inner compartment of the BS, where they are decarboxylated to release CO_2_ to the Calvin cycle (Hatch, 1987; Furbank and Taylor, 1995; Furbank *et al.*, 2004). The mechanism of decarboxylation in BS cells has been traditionally divided into three different C_4_ types; NAD-malic enzyme (NAD-ME), NADP-malic enzyme (NADP-ME), and phosphoenolpyruvate carboxykinase (PEPCK) (Hatch, 1987), with some degree of flexibility or co-existence (Wang *et al.*, 2014) (Fig. 1). However, only NAD-ME and NADP-ME subtypes are considered to be exclusive subtypes from specific lineages; both contain the PEPCK cycle as the supplementary photosynthetic pathway (Wang *et al.*, 2014; Liu and Osborne, 2015).

C_4_ photosynthesis is thought to have first arisen ~30 million years ago and is found in >66 independent lineages of monocotyledons and dicotyledons (Aubry *et al.*, 2014; Burgess and Hibberd, 2015) (Fig. 1). Most of the C_4_ species occur in the grasses (~4600) and sedges (~1600) of monocots, while only 1600 C_4_ species are found in the dicots (Gowik and Westhoff, 2011). The mechanism of establishment of C_4_ photosynthesis is still unclear. Genes involved in C_4_ photosynthesis recruited existing C_3_ chloroplastic ancestors and did not evolve *de novo* (Gowik and Westhoff, 2011; Maier *et al.*, 2011). C_4_ photosynthesis may have emerged through gene duplication, promoter insertion, alteration of leaf structure, and establishment of the photorespiratory CO_2_ pump (Gowik and Westhoff, 2011; Maier *et al.*, 2011).

A distinct set of transcripts are preferentially expressed in the fully specialized M or BS cells to enable their co-operation in carbon fixation (Majeran *et al.*, 2005). Comparison of transcriptomes for M and BS cell-specific gene expression in different lineages of C_4_ plants will provide insight into the commonality and differentiation of regulatory mechanisms for the compartmentalization of C_4_ photosynthesis in its independent evolutionary origins (Burgess and Hibberd, 2015). Only limited lineages have so far been assessed for M and BS cell-specific profiles; two for the monocot NADP-ME subtype and one for the dicot NAD-ME subtype. Chang *et al.* (2012) and Li *et al.* (2010) generated M and BS transcriptome profiles from mature leaves of maize, a monocot NADP-ME subtype. Pairwise comparisons of M and BS transcriptomes have been conducted for maize versus *Setaria viridis* (monocot NADP-ME subtype), and maize versus *Cleome gynandra* (dicot NAD-ME subtype) (Li *et al.*, 2010; Chang *et al.*, 2012; Aubry *et al.*, 2014; John *et al.*, 2014). However, the transcript expression profiles of M and BS cells in a monocot NAD-ME subtype C_4_ plant have yet to be determined, resulting in the absence of a global comparison of cell-specific transcriptomes in the two distinct subtypes of monocot and dicot C_4_ plants.

Switchgrass (*Panicum virgatum* L.) is being targeted as a source of biomass for biofuel production (McLaughlin and Kszos, 2005; Bouton, 2007), and is here selected as the representative of the monocotyledonous NAD-ME-type C_4_ plant (Christin *et al.*, 2009). We first defined the transcriptomic profiles of M and BS cells from switchgrass by manually isolating M and BS cells from mature leaves. We further applied comparative transcriptome analysis to dissect the evolutionary convergence and divergence of monocot and dicot C_4_ plants: switchgrass, maize, *Setaria viridis*, and *Cleome gynandra*. Our study provides an overview of the functional differentiation and co-ordination of M and BS cells in the C_4_ photosynthesis pathway, and provides insights into the possible evolutionary pathway of differentiation in C_4_ photosynthesis.

## Materials and methods

### Plant material, growth conditions, and enzyme assays

Switchgrass plants (cultivar Alamo-AP13) were grown in the greenhouse at 28 °C and 16/8h day/night conditions, using supplemental lighting from halide lamps (250mol photons m^−2^ s^−1^). For age gradient experiments, leaves were harvested from the first fully expanded leaves to the fourth leaves of three-node stage plants. For leaf developmental gradient experiments, leaves were harvested as the top, middle, and base sections from the second or third leaf of three-node stage plants. Samples were immediately frozen in liquid nitrogen and extracted as described (Sommer *et al.*, 2012). Enzyme activity measurements of NAD-ME, NADP-ME, PEPCK, aspartate aminotransferase (AspAT), and alanine aminotransferase (AlaAT) were conducted using coupled spectrophotometric assays as described previously (Sommer *et al.*, 2012). All assays were performed at 28 °C. The concentration of total soluble protein was determined by the Bradford method (Bradford, 1976) using the Bio-Rad protein assay reagent at 595nm.

### Fixation, cryosectioning, and cell-specific micro-dissection

The middle sections from the second or third leaf of three-node stage plants were fixed and subsequently used for micro-dissection. A Leica CM 1850 cryostat (Leica Microsystems Inc., Bannockburn, IL, USA) was used for obtaining cross-sections of switchgrass leaves, which were processed by fixation and cryoprotection as described previously (Srivastava *et al.*, 2010). We found that the optimum longitudinal section thickness was 10 μm. For obtaining M and BS cells, leaf sections were manually dissected with a scalpel blade under a fluorescence stereo microscope. Around 1000 cells were collected into each tube and stored at −80 °C, and every 5–10 tubes were used together for RNA isolation.

### RNA extraction and analysis

Total RNA was extracted from manually harvested M and BS cells using an RNeasy Micro Kit (Qiagen, Valencia, CA, USA) according to the manufacturer’s protocol. RNA quality and quantity were estimated using an RNA 6000 Pico chip on an Agilent 2100 Bioanalyzer (Agilent Technologies, Palo Alto, CA, USA). RNA was then amplified using an Arcturus^®^ RiboAmp^®^ HS PLUS 2-round kit (Life Technologies, CA, USA) following the manufacturer’s protocol (Li *et al.*, 2010). At least 1ng of starting total RNA was used for amplification of each replicate. The quality of amplified RNA was checked with the Bioanalyzer 2100, using the RNA 6000 nanochip.

### Construction of cDNA libraries and sequencing procedure

As previously described (Rao *et al.*, 2014), 1 μg of total RNA for each sample was used to construct an RNA sequencing (RNA-seq) library using TruSeq RNA Sample Prep Kits v2 (Illumina Inc., San Diego, CA, USA), according to the manufacturer’s instructions, at the Genomics Core Facility at the Noble Foundation (Ardmore, OK, USA). Each library was indexed. Six libraries with different indexes were pooled together in one lane for 100bp paired-end sequencing. The Hiseq2000 run was conducted at the Genomics Core Facility of the Oklahoma Medical Research Foundation (Oklahoma City, OK, USA).

### Determination of transcript abundance

All paired-end Illumina reads were trimmed using in-house PERL scripts with two filters, quality scores of ≤20 from the end of each read, and poly(A) (forward sequencing) or poly(T) (reverse sequencing) tails >15bp in length. Reads <50bp in length after trimming were discarded along with their mates. The trimmed reads were mapped to the Switchgrass genome *Panicum virgatum* v1.1 (http://phytozome.jgi.doe.gov/) using Bowtie 2 version 2.0.0 (Langmead and Salzberg, 2012) and TopHat version 2.0.10 (Kim *et al.*, 2013) in conjunction with SAMtools version 0.1 (Li *et al.*, 2009) with default parameters. Based on *Panicum virgatum* v1.1 annotation, normalized gene expression levels were calculated in FPKM (fragments per kilobase of exon model per million mapped fragments) (Trapnell *et al.*, 2010) using Cufflinks version 2.1.1 (default settings, set to 100 mean inner distance for paired-end reads; Trapnell *et al.*, 2013) and in TPM (transcripts per million) (Wagner *et al.*, 2012) using RSEM version 1.2.23 (default settings; Li and Dewey, 2011). For differential expression testing, alignments of reads from Tophat v2.0.10 were counted to genes using HT-SEQ with union mode (Anders *et al.*, 2015), then analyzed using DESeq2 (Love *et al.*, 2014). A Benjamini–Hochberg corrected *P*-value of <0.05 was set to identify differentially expressed genes. Raw data are provided in Supplementary Table S1 at *JXB* online.

### Data annotation

*Panicum virgatum* v1.1 gene annotation was downloaded from the Phytozome v10.3 website (http://phytozome.jgi.doe.gov/). Classification for cell type-enriched genes was based on MapMan mappings of their Arabidopsis homologs (Thimm *et al.*, 2004). Significant functional enrichment was determined using Fisher’s exact test with Benjamini–Hochberg multiple testing correction [false discovery rate (FDR) ≤0.1]. Identification of transcription factors (TFs) in *Panicum virgatum* v1.1 genes was based on their Arabidopsis and rice homologs and the annotation from the Plant Transcription Factor Database v3.0 (Jin *et al.*, 2014). Identification of homologous genes among switchgrass, maize, and *S. viridis* was performed using alignments of *Panicum virgatum* v1.1, *Setaria italica* v2.1, and *Zea mays* v5b.60 protein sequences obtained from Phytozome v10.3 using blast+ tools. The syntenic gene set of maize, sorghum, and rice was obtained from Schnable *et al.* (2012) and that of switchgrass, *S. viridis*, and maize was generated for pairwise comparisons using SynMap (Lyons *et al.*, 2008) with default parameters (score ≥90).

### Real-time quantitative reverse transcription–PCR

Total RNA was isolated from the second and third leaf, leaf sheath, and stem of three-node stage switchgrass plants. cDNA synthesis, primer design, and real-time quantitative reverse transcription–PCRs (qRT–PCRs) were performed as previously described (Rao *et al.*, 2014). qRT–PCR was performed in triplicate for each sample, and three biological replicates were evaluated for each gene tested. Data were collected and analyzed using PikoReal Software (Thermo Scientific). PCR efficiency was estimated using PikoReal software, and transcript levels were determined by relative quantification using the actin gene as a reference (Gimeno *et al.*, 2014).

### *In situ* hybridization

For *in situ* hybridization, samples of middle sections from the second or third leaf of three-node stage switchgrass plants were harvested. Tissue fixation, dehydration, and paraffin embedding were performed according to Long’s protocol (http://www.its.caltech.edu/~plantlab/protocols/insitu.pdf). Pre-hybridization, hybridization, and washing were conducted on the robotic GenePaint™ system (Tecan Inc., Durham, NC, USA) following the manufacturer’s instructions (Zhou *et al.*, 2011).

### Construction of phylogenetic trees

For phylogenetic reconstruction, multiple protein sequences were aligned using the ClustalW algorithm (Larkin *et al.*, 2007). Neighbor–Joining (NJ) phylogenetic trees were generated by the MEGA 6.0 program (Tamura *et al.*, 2013), using 1000 replicates of bootstrap analysis. Protein sequences were obtained from a previous report (Maier *et al.*, 2011).

### Accession numbers

The data sets supporting the results of this article are available in the NCBI Sequence Read Archive (SRA) repository, NCBI SRA accession no. SRX1160366.

## Results and Discussion

### Activity of C_4_ enzymes in the switchgrass leaf

**Fig. 2.**
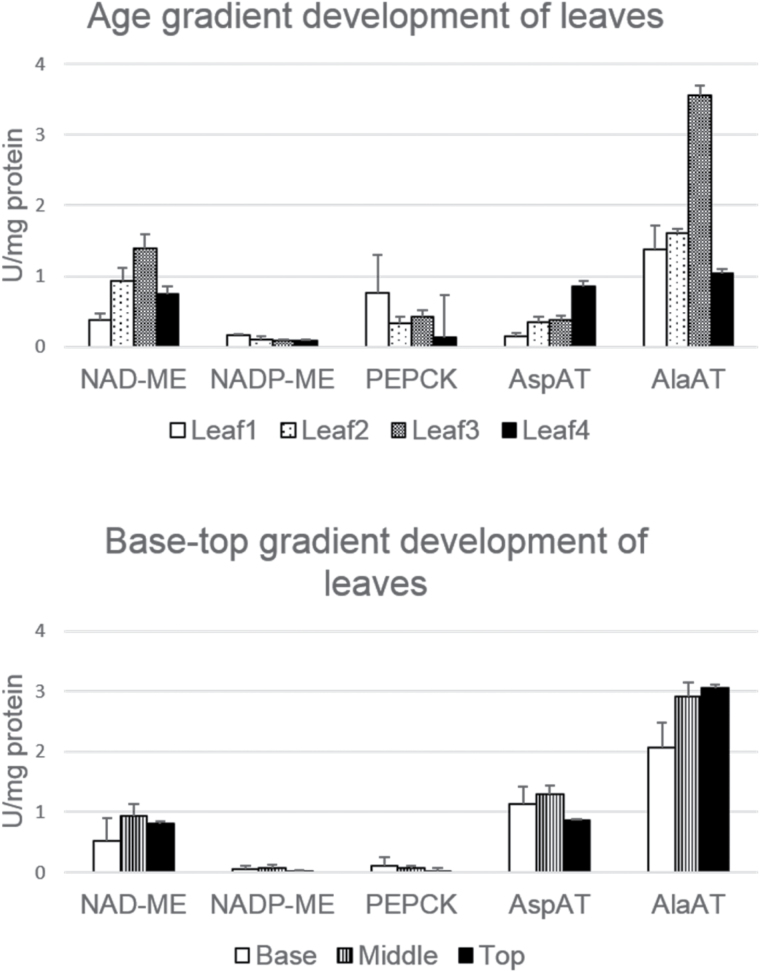
Enzyme activities in age and base–top gradient development of switchgrass leaves. One unit (U) of enzyme activity is defined as the amount of enzyme required to produce 1.0 µmol of product in 1min. Means and standard errors from three independent biological replicates are plotted. The significant differences by the Student’s *t*-test between pairwise samples are provided in Supplementary Table S2 at *JXB* online.

To determine the differences in CO_2_ fixation enzymes along a developmental gradient in switchgrass leaves as a basis for selection of tissues for transcriptomic analysis, we measured the activities of enzymes involved in C_4_ photosynthetic pathways in crude leaf extracts. For the age gradient experiment, we selected from the first to the fourth leaf blades as young, mature, and old samples from plants with three nodes, in the elongation stage of the plant (Moore *et al.*, 1991). The three decarboxylating enzymes NAD-ME, NADP-ME, and PEPCK were assayed, as well as AspAT and AlaAT (Fig. 2; Supplementary Table S2). This analysis confirmed that switchgrass performs C_4_ photosynthesis of the NAD-ME type with a minimal PEPCK decarboxylating pathway (Warner *et al.*, 1987). Compared with young and old samples, the mature leaves (the second and third leaves) showed significantly higher activity of NAD-ME, AspAT, and AlaAT. A base to top gradient experiment was further conducted on the second and the third leaves to assess area-related differences in C_4_ enzyme activities of mature leaves. The classical NAD-ME subtype enzymes NAD-ME and AlaAT showed lower activities in the base section of leaves compared with the top or middle sections. This is consistent with the results of transcriptomic and C_4_ enzyme activity assays in maize leaves, since cells in the leaf base are younger with minimal photosynthetic activity (Li *et al.*, 2010; Pick *et al.*, 2011). Considering the C_4_ enzyme activity and the size of the leaf cells, the middle sections in the mature zones of the second and third leaves were selected for subsequent experiments.

### Isolation of bundle sheath and mesophyll cells

Cross-sections from selected parts of the switchgrass leaf showed the typical Kranz anatomy structure of NAD-ME subtype C_4_ plants; a layer of small M cells surrounding the layer of large BS cells with the inner mestome sheath and vascular tissue (Supplementary Fig. S1) (Edwards and Walker, 1983; Edwards *et al.*, 2001). As observed in a previous study (Warner *et al.*, 1987), the >10 μm width of BS and M cells makes hand dissection feasible (Supplementary Fig. S1). Hand dissection has been successfully applied for tissue-specific and single-cell analysis in plant research, and can be simply carried out by use of a razor blade (Outlaw and Zhang, 2001). In this study, leaf fragments were cut into longitudinal cryosections with optimal thickness of 10 μm, and BS and M cells were obtained by manual micro-dissection under a fluorescence stereo microscope (Supplementary Fig. S1). RNA isolated from BS and M cells (Supplementary Fig. S2) was of suitable quality for amplification, library construction, and sequencing.

### Deep sequencing, read mapping, and identification of expressed genes

Duplicated BS and M cell samples from switchgrass leaves were sequenced on the HiSeq™ Sequencing System to generate paired-end reads. Clean sequence reads for BS and M samples were mapped to the switchgrass reference genome *Panicum virgatum* v1.1, resulting in 79% of the cleaned reads mapped to the reference genome (Table 1). Considering the high complexity of the tetraploid switchgrass genome (Sharma *et al.*, 2012) and the fact that *Panicum virgatum* v1.1 represents a partial genome sequence, a moderate ratio of mapped reads is expected.

**Table 1.** Statistics of the transcriptome data

**Parameter**	**Value**
Read length	100 bp
Read type	Paired
Total reads	144 129 974
Trimmed reads	111 728 008
Mapped reads	87 920 267 (79%)
Detected genes (mapped read >1)	46 716
Differentially expressed genes	9752

Normalized transcript levels were calculated using RSEM version 1.2.23 (Li and Dewey, 2011) and Cufflinks version 2.1.1 (Trapnell *et al.*, 2013) in terms of TPM (Wagner *et al.*, 2012) and FPKM (Trapnell *et al.*, 2010). To define ‘differentially expressed genes’, we used the criterion of adjusted *P*≤0.05 between the two RNA samples. On this basis, 5122 and 4630 genes were considered as differentially expressed in BS and M cells, respectively. Moreover, increasing the criterion of difference in fold value, more genes were enriched in BS cells than in M cells (Table 2). For example, 332 genes in BS but only 199 genes in M showed a 16-fold difference in expression level. This finding is consistent with observations in the other C_4_ plants maize (Chang *et al.*, 2012) and *S. viridis* (John *et al.*, 2014), and suggests that BS cells play a more important role in C_4_ photosynthesis and may also differentially perform other metabolic processes (Chang *et al.*, 2012).

**Table 2.** Numbers of expressed genes, differentially expressed genes, and TF genes in BS and M samples

**Parameter**	**BS cells**	**M cells**
Differentially expressed genes (adjustws *P*<0.05)	5,122	4,630
Differentially expressed genes (ratio >2)	4,343	3,886
Differentially expressed genes (ratio >4)	2,444	2,126
Differentially expressed genes (ratio >8)	923	695
Differentially expressed genes (ratio >16)	332	199
Differentially expressed TF genes	175	243

### Estimation of C_4_ pathway transcript abundance in the switchgrass leaf

The main difference in the photosynthetic pathway between C_3_ and C_4_ plants is that in C_4_ plants the CO_2_ concentration and assimilation mechanisms are divided between M and BS cells (Chang *et al.*, 2012). Gene families that encode proteins with key roles in C_4_ photosynthesis were detected in our M/BS transcriptome data set (Supplementary Table S3). Among them, 25 genes were selected for further analysis due to strong cell-specific expression (Table 3). The fold change between M and BS samples was similar using either the FPKM or TPM value (Supplementary Table S3). Because TPM eliminates statistical biases compared with FPKM (Wagner *et al.*, 2012), we used TPM to present expression levels of C_4_ genes in the following sections.

**Table 3.** Differential expression of C_4_ photosynthesis-specific genes between M and BS cells

**Gene**	**Role**	**Cell type**	**ID**	**M (TPM**)	**BS (TPM**)	**Log**_**2**_ **(M/BS)**	**Adjusted *P***
CA	Core pathway	M	Pavir.J05107	10360.08	2559.41	1.74	0.0043498
PEPC	Core pathway	M	Pavir.Da00871	404.89	132.94	1.35	0.0158103
PPDK	Core pathway	M	Pavir.J15849	10630.50	1774.50	2.24	1.567E-09
ASP-AT(M)	Core pathway	M	Pavir.Eb03232	2699.88	559.12	1.79	5.627E-07
ASP-AT(BS)	Core pathway	BS	Pavir.J06248	91.64	1045.03	–3.27	1.32E-17
ALA-AT	Core pathway	BS/M	Pavir.Ba00407	4.82	17.78	–2.09	5.823E-08
NADP-MDH	Core pathway	M	Pavir.Fa00047	193.63	39.84	1.71	4.326E-07
NADP-ME	Core pathway	BS	Pavir.Eb00308	188.46	4081.74	–4.58	3.438E-53
NAD-ME	Core pathway	BS	Pavir.Ia01553	18.64	695.89	–4.84	8.162E-89
NAD-MDH	Core pathway	BS	Pavir.J08443	108.92	1566.72	–4.35	0
PEPCK	Core pathway	BS	Pavir.Ia03881	9.77	59.16	–2.56	5.193E-06
RBCS	Calvin cycle	BS	Pavir.Ca02105	279.46	8643.50	–5.18	0
RCA	Calvin cycle	BS	Pavir.Hb00014	23.07	627.48	–4.64	8.738E-75
PRK	Calvin cycle	BS	Pavir.Aa01018	68.43	1903.17	–4.86	1.096E-41
CP12	Calvin cycle	BS	Pavir.J38955	0.85	19.27	–3.91	1.398E-11
RPI	Calvin cycle	BS	Pavir.Bb00418	130.41	2702.07	–4.52	1.1E-171
RPE	Calvin cycle	BS	Pavir.Ia04312	133.34	3492.42	–4.72	1.788E-31
TKL	Calvin cycle	BS	Pavir.Da02287	44.74	1504.46	–5.28	1.22E-242
SBP	Calvin cycle	BS	Pavir.J03580	6.71	287.19	–4.91	2.529E-50
FBP	Calvin cycle	BS	Pavir.J33737	7.74	81.39	–3.36	5.467E-24
FBA	Calvin cycle	BS	Pavir.J02023	56.93	1424.97	–4.53	1.08E-244
PGK	Calvin cycle	M	Pavir.J00566	5471.47	1559.81	1.33	0.0048193
GADPH(A)	Calvin cycle	BS	Pavir.Ca01865	30.68	54.80	–1.07	0.0002747
GADPH(B)	Calvin cycle	M	Pavir.J23272	3301.02	1316.49	1.17	4.605E-14
TPI	Calvin cycle	M	Pavir.J26504	815.95	108.90	2.62	1.611E-33

CA, carbonic anhydrase; PEPC, phosphoenolpyruvate carboxylase; PPDK, pyruvate/orthophosphate dikinase; AspAT, aspartate aminotransferase; AlaAT, alanine aminotransferase; NADP-MDH, NADP-dependent malate dehydrogenase; NADP-ME, NADP-dependent malic enzyme; NAD-MDH, NAD-dependent malate dehydrogenase; NAD-ME, NAD-dependent malic enzyme; PEPCK, phosphoenolpyruvate carboxykinase; RBCS, ribulose bisphosphate carboxylase small chain; RCA, Rubisco activase; PRK, phosphoribulokinase; CP12, Calvin cycle protein CP12; RPI, ribose 5-phosphate isomerase; RPE, ribulose-phosphate 3-epimerase; TKL, transketolase; SBP, sedoheptulose-bisphosphatase; FBP, fructose-1,6-bisphosphatase; FBA, fructose-bisphosphate aldolase; PGK, phosphoglycerate kina; GADPH, glyceraldehyde-3-phosphate dehydrogenase; TPI, triosephosphate isomerase.

The C_4_ marker genes encoding carbonic anhydrase (CA), pyruvate orthophosphate dikinase (PPDK), PEPC, and NAD-dependent malate dehydrogenase (NADP-MDH; Fig. 1B) that are involved in carbon fixation (Chang *et al.*, 2012) were preferentially expressed in M cells, whereas NAD-MEs, NAD-MDH, and PCK that are involved in releasing CO_2_ from C_4_ acid (Chang *et al.*, 2012), and nine genes encoding enzymes involved in the Calvin cycle, showed much higher transcript levels in BS cells than in M cells. In addition, phosphoglycerate kinase (PGK), glyceraldehyde 3-phosphate dehydrogenase B subunit (GADPH-B), and triosephosphate isomerase (TPI), that work in M cells to allow balancing of reducing equivalents between the two cell types (John *et al.*, 2014), were preferentially expressed in the M cells.

**Fig. 3.**
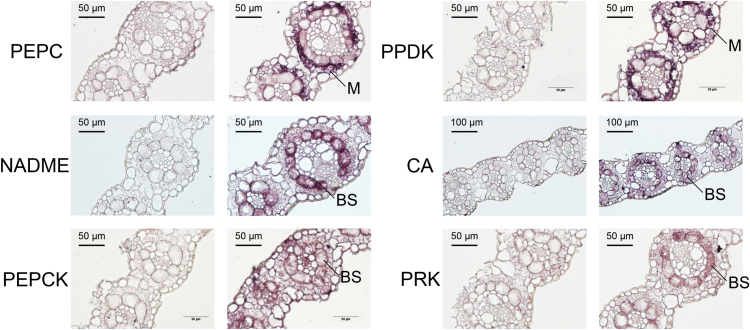
*In situ* hybridization of C_4_-related gene transcripts in mature leaves. (This figure is available in colour at *JXB* online.)

On the basis of previously published microarray analyses (Zhang *et al.*, 2013), C_4_ marker genes in switchgrass displayed a higher expression level in leaf blade compared with other major tissues and organs such as leaf sheath and stem (Supplementary Fig. S3). The high expression of C_4_ marker genes in the mature switchgrass leaf was confirmed through qRT–PCR analysis (Supplementary Fig. S3). To validate further the digital expression profile, NAD-ME, CA, PEPCK, phosphoribulokinase (PRK), PEPC, and PPDK were selected for localization analysis by *in situ* hybridization. Labeled antisense probes were used to hybridize with the target mRNAs *in situ*, while labeled sense probes were used as negative controls (Srivastava *et al.*, 2010). The sequences of the primers used for qRT–PCR and *in situ* hybridization are given in Supplementary Table S4. As expected, NAD-ME, CA, PEPCK, and PRK were preferentially expressed in BS cells, whereas PEPC and PPDK transcripts were enriched in M cells (Fig. 3). These results are consistent with those from the transcriptome data set, and indicate that our isolation methods caused only low-level cross-contamination of M and BS cells.

### Convergence in C_4_ transcript abundance among monocot and dicot C_4_ plants

**Fig. 4.**
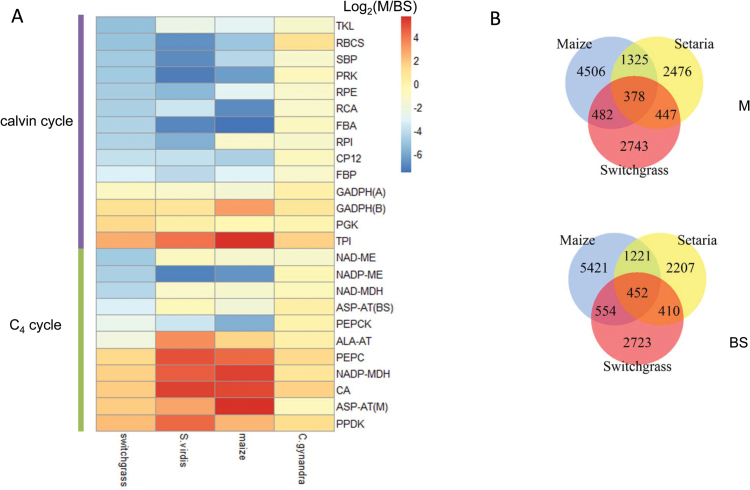
Global comparison of transcript abundance within M and BS cells of four C_4_ species. (A) Fold change of expression level of genes involved in C_4_ metabolism and the Calvin cycle in M and BS cells from switchgrass, *S. viridis*, maize, and *C. gynandra*. (B) Venn diagrams show the number of homologous genes differentially expressed in M and BS cells from switchgrass, *S. viridis*, and maize. (This figure is available in colour at *JXB* online.)

We compared C_4_ transcript abundance in the M and BS transcriptomes from switchgrass (present work), *S. viridis* (John *et al.*, 2014), maize (Chang *et al.*, 2012), and *C. gynandra* (Aubry *et al.*, 2014) (Fig. 4; Supplementary Table S5). Maize, *S. viridis*, and switchgrass belong to the C_4_ grasses, and are classified into two distinct subtypes, NADP-ME and NAD-ME (Wang *et al.*, 2014). The dicot plant *C. gynandra* performs NAD-ME-type C_4_ photosynthesis (Sommer *et al.*, 2012). The major biochemical processes for C_4_ carbon fixation in the two C_4_ photosynthetic subtypes are shown in Fig. 1B. Transcripts involved in the C_4_ cycle showed very similar compartmentalization between M and BS cells in the grass species; genes encoding enzymes involved in CO_2_ fixation (CA, PEPC, PPDK, and NADP-MDH) and the Calvin cycle [ribulose bisphosphate carboxylase small chain (RBCS), Rubisco activase (RCA), PRK, ribose 5-phosphate isomerase (RPI), ribulose-phosphate 3-epimerase (RPE), transketolase (TKL), sedoheptulose-bisphosphatase (SBP), fructose-1,6-bisphosphatase (FBP), and fructose-bisphosphate aldolase (FBA)] were enriched in M and BS cells, respectively. Transcripts from the *C. gynandra* transcriptome showed reduced enrichment in BS cells, possibly due to post-transcriptional regulation (Aubry *et al.*, 2014). The consistency of C_4_ core gene expression preference in four distinct C_4_ lineages highlights the evolutionary convergence in the C_4_ pathway.

The GADPH of land plants includes two plastidic tetramer isoforms named A_4_ and A_2_B_2_ (Fermani *et al.*, 2012). Transcripts encoding GADPH-B were clearly enriched in M cells in the four species, consistent with the role of the A_2_B_2_ isoform in catalyzing the reducing step of the Calvin cycle in M cells. In contrast, transcripts encoding GADPH-A accumulated in both cell types, with a slightly higher ratio in BS cells. Furthermore, transcripts encoding CP12, a small protein involved in regulation of the Calvin cycle by forming a supercomplex with PRK and GADPH (Groben *et al.*, 2010; Lopez-Calcagno *et al.*, 2014), were exclusively found in BS cells. This suggests that in BS cells, CP12 and A_4_ GADPH mainly function together for regulation of the activity of PRK, in agreement with the observation of formation of a stable CP12–A_4_ GADPH complex in the dark (Fermani *et al.*, 2012).

PEPCK, the decarboxylation enzyme that utilizes OAA to release CO_2_ in the cytosol (Hatch, 1987), is enriched in BS cells at moderate expression levels in all four C_4_ species. This is consistent with the detection of PEPCK activity in switchgrass, maize, and *C. gynandra* (Prendergast *et al.*, 1987; Sommer *et al.*, 2012), and suggests that a mixed and flexible decarboxylation system commonly exists in NAD-ME and NADP-ME subtypes, through an additional service of the PEPCK cycle (Furbank, 2011; John *et al.*, 2014).

### Divergence in C_4_ transcript abundance between NAD-ME and NADP-ME subtypes

C_4_ plants have evolved different means to decarboxylate organic carbon compounds to release CO_2_ at the site of Rubisco; NADP-ME decarboxylates malate to pyruvate in chloroplasts, whereas NAD-ME decarboxylates malate to pyruvate in mitochondria (Brautigam *et al.*, 2014) (Fig. 1B). Transcripts encoding NADP-ME, rather than NAD-ME or NAD-MDH, show enrichment in BS cells in two different lineages of the NADP-ME subtype (maize and *S. viridis*), whereas NAD-ME is enriched in BS cells in the monocot switchgrass and the dicot *C. gynandra* (Fig. 4). In the phylogenetic tree constructed with known NAD-ME sequences, four switchgrass NAD-ME genes detected in our M/BS transcriptome data set are classified into two distinct clusters, and photosynthetic NAD-ME in switchgrass belongs to the NAD-ME 2 group (Supplementary Fig. S4).

Interestingly, transcripts of two switchgrass genes (PvEa00263 and PvEb00308) that are annotated as chloroplastic NADP-ME showed high accumulation in BS cells. The high expression level of NADP-ME genes in switchgrass leaves has been observed in several previous reports (Zhang *et al.*, 2013; Meyer *et al.*, 2014; Palmer *et al.*, 2015) and here was confirmed by qRT–PCR analysis using multiple pairs of primers (Supplementary Fig. S3; Supplementary Table S4). To address the evolutionary origin of switchgrass NADP-MEs, a phylogenetic tree was constructed from protein sequence alignments of NADP-MEs from C_4_ and C_3_ plants (Supplementary Fig. S5). The two highly expressed switchgrass NADP-ME genes clustered with the C_4_ NADP-ME of *S. viridis*, and share identity to the non-photosynthetic plastidic NADP-ME in maize (Saigo *et al.*, 2004), which presents high intrinsic NADP-ME activity but is expressed constitutively at low levels (Maier *et al.*, 2011). Considering the low NADP-ME activity detected in switchgrass leaves, it is likely that in switchgrass either strong post-transcriptional or translational control leads to degradation or loss of activity of NADP-ME in BS cells, or the two genes encode low activity NADP-ME isoforms. To assess further the possible derivation of C_4_ functional decarboxylation enzyme from the same ancestral genomic region (Schnable *et al.*, 2012), we identified the syntenic orthologs of NAD-ME and NADP-ME isoforms among C_4_ grasses and outgroup C_3_ rice (Supplementary Fig. S6). Interestingly, NAD-ME 2 genes (functional C_4_ group) were syntenic within C_4_ grasses (switchgrass, *S. viridis*, maize, and sorgum), whereas NAD-ME 1 genes were syntenic between C_4_ grasses and C_3_ rice. Genes in non-C_4_ and C_4_ NADP-ME groups were syntenic in switchgrass, *S. viridis*, maize, and sorghum. Overall, it is possible that the photosynthetic and non-photosynthetic isoforms found in C_4_ plants could present at some level within the most recent common ancestor (Washburn *et al.*, 2015) but may or may not have been recruited into C_4_ photosynthesis depending on selective pressure.

In NAD-ME and PEPCK subtypes, carboxylation and decarboxylation are linked by the transfer of aspartic acid and alanine in M/BS cells, whereas NADP-ME subtypes mainly recruit malate/pyruvate shuttles between the two cell types (Weber and von Caemmerer, 2010). The amino acid aspartate in the chloroplast of M cells is generated by chloroplastic AspAT, then enters the BS cells where it is converted into OAA in the mitochondria by mitochondrial AspAT (Weber and von Caemmerer, 2010) (Fig. 1B). In switchgrass, transcripts encoding chloroplastic and mitochondrial AspAT are abundant in M and BS cells, respectively, which supports a function in maintaining the ammonia balance between M/BS cell types (Weber and von Caemmerer, 2010). In contrast, AspAT shows reduced expression in BS cells in the NADP-ME subtypes maize and *S. viridis*. Transcripts encoding AlaAT are significantly accumulated in both M and BS cells of *C. gynandra*, consistent with a dual function in converting from/into alanine in M/BS cells. In contrast, the AlaAT in the NADP-ME subtypes (maize and *S. viridis*) displayed unequal distribution in M and BS cells. However, the apparent low expression level of the AlaAT gene in switchgrass is possibly artifactual due to the incompleteness of the switchgrass genome. At least 20 expressed sequence tags (ESTs) were predicted to represent genes encoding AlaAT in switchgrass (Wang *et al.*, 2012; Zhang *et al.*, 2013); one EST (AP13CTG00178) was highly expressed in mature leaves with similar expression pattern to other C_4_ genes (SupplementaryFig. S3), but is not represented in the current genome assembly *Panicum virgatum* v1.1.

### Differences in C_4_ shuttle/transport between NAD-ME and NADP-ME subtypes

**Fig. 5.**
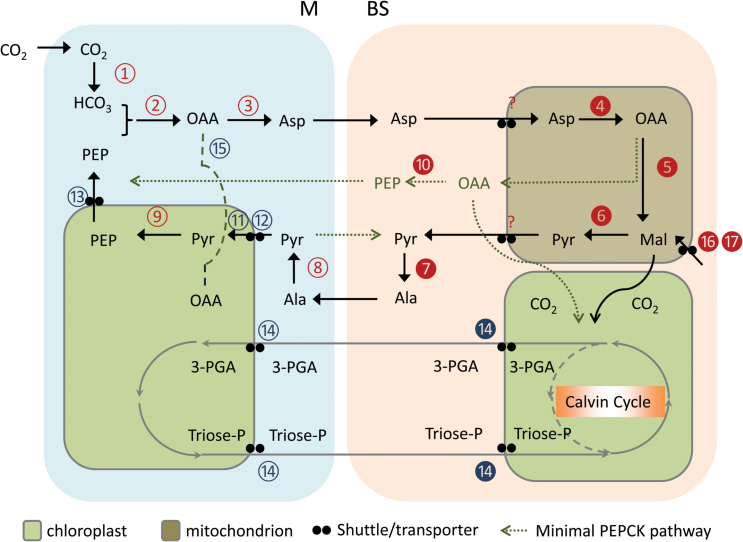
Model for NAD-ME with minimal PEPCK pathway. 1, carbonic anhydrase (CA); 2, phosphoenolpyruvate carboxylase (PEPC); 3, chloroplastic aspartate aminotransferase (AspAT); 4, mitochondrial aspartate aminotransferase (AspAT); 5, NAD-dependent malate dehydrogenase (NAD-MDH); 6, NAD-dependent malic enzyme (NAD-ME); 7, alanine aminotransferase (AlaAT) in bundle sheathes cells; 8, alanine aminotransferase (AlaAT) in mesophyll cells; 9, pyruvate/orthophosphate dikinase (PPDK); 10, phosphoenolpyruvate carboxykinase (PEPCK); 11, sodium bile acid symporter (BASS) homolog; 12, sodium: hydrogen antiporter (NHD); 13, phosphate/phosphoenolpyruvate translocator (PPT); 14, triose phosphate/phosphate translocator (TPT); 15, dicarboxylate transporter (DiT1); 16, malate phosphate antiporter (DIC); 17, phosphate/protons symporter (PIC). Ala, alanine; Asp, aspartate; Mal, malate; Pyr, pyruvate; OAA, oxaloacetate; PEP, phosphoenolpyruvate; 3-PGA, 3-phosphoglycerate, Triose-P, triose phosphate. (This figure is available in colour at *JXB* online.)

C_4_ photosynthesis recruits a high rate of metabolic exchange between the two main cell types, which requires multiple transporters in M and BS chloroplast envelopes to facilitate this metabolic collaboration (Majeran and van Wijk, 2009). Based on the present study and previous work (Majeran and van Wijk, 2009; Weber and von Caemmerer, 2010; Brautigam *et al.*, 2014), the detailed processes of C_4_ photosynthesis in the NAD-ME subtype are summarized in Fig. 5.

For both NAD-ME-type and NADP-ME-type C_4_ plants, pyruvate is used to regenerate the acceptor PEP in M cells and is thereby required to transfer from the cytosol to the chloroplast. However, plants may evolve different strategies to pump pyruvate into these two subtypes (Majeran and van Wijk, 2009). In switchgrass, we observed high enrichment of transcripts corresponding to two genes (Pv.Ea02237 and Pv.Eb01809) encoding the plastidic sodium-dependent pyruvate transporter BASS2 (Furumoto *et al.*, 2011) and moderate enrichment of transcripts corresponding to a plastidic proton:sodium symporter NHD1 gene (Pv.Ba03078) in M cells. The preferential enrichment of BASS2 and NHD1 transcripts in M cells was also found in the NAD-ME subtype *C. gynandra* (BADSS2, AT2G26900; and NHD1, AT3G19490), but not in maize and *S. viridis* which, as NADP-ME subtype plants, use the sodium/pyruvate symporter (Chang *et al.*, 2012). This supports the hypothesis that in NAD-ME subtype plants, sodium-dependent transporters take pyruvate into M cell chloroplasts and NHD1 exports sodium from the chloroplast in order to maintain the sodium gradient (Brautigam *et al.*, 2011) (Fig. 5).

In both NAD-ME and NADP-ME subtype plants, PEP generated from pyruvate in M cell chloroplasts is exported into the cytosol by a PEP/phosphate translocator (PPT) (Brautigam *et al.*, 2011). Similarly in maize and *S. viridis* (Chang *et al.*, 2012), two transcripts (Pv.Fa00799, M TPM=913; and Pv.Fb00666, M TPM=650) encoding PPT were enriched in M cells, whereas the other three (Pv.J33600, Pv.Ea00394, and Pv.J15038) were enriched in BS cells. This suggests that PPT encoded by Pv.Fa00799 and Pv.Fb00666 may specifically export PEP and import triose phosphate in the M chloroplasts. The triose phosphate translocator (TPT) is thought to export triose phosphates and 3-phosphoglycerate (3-PGA) from chloroplasts to the cytosol in M and BS cells; this is necessary to generate half reducing equivalents for the Calvin cycle (Knappe *et al.*, 2003; Weber and von Caemmerer, 2010). In switchgrass, maize, and *S. viridis* (Chang *et al.*, 2012), genes encoding TPT were expressed at a high level in both M and BS cells. In *C. gynandra*, PPT (AT5G33320) and TPT (AT5G46110) display high expression levels in both cell types as well. This suggests that C_4_ plants recruit PPT and TPT to transfer PEP/phosphate and triose phosphate, respectively, in both NAD-ME and NADP-ME subtypes.

For NADP-ME type C_4_ photosynthesis, DiT1 (a 2-oxoglutarate/malate transporter) exchanges OAA/malate across the M cell chloroplast envelope membrane and DiT2 imports malate into BS chloroplasts (Majeran and van Wijk, 2009; Chang *et al.*, 2012). These processes are not required for NAD-ME-type C_4_ photosynthesis (Brautigam *et al.*, 2011). Genes encoding DiT1 were preferentially expressed in M cells and genes encoding DiT2 were only weakly expressed in both M and BS cell types in switchgrass (Supplementary Table S6). No increased transcript abundance of DiT1 in C_4_ (*C. gynandra*) was detected compared with its closely related C_3_ species (*C. spinosa*) (Brautigam *et al.*, 2011). This suggests that DiT1 may not act specifically for C_4_ metabolite transport in M cells in the NAD-ME subtype because it dually acts as the malate valve and as a 2-oxoglutarate/malate transporter for photorespiratory nitrogen recycling (Kinoshita *et al.*, 2011). For NAD-ME-type C_4_ photosynthesis, malate is required to be generated from OAA in mitochondria or transported into mitochondria (Fig. 5). We found that transcripts encoding the mitochondrial malate/phosphate carrier DIC (Pavir.Fb01780) and the mitochondrial phosphate transporter PIC2 (Pavir.Aa00590) were significantly enriched in switchgrass BS cells with a high expression level. Similar BS cell-type enrichment and high expression of genes encoding two mitochondrial transporters (DIC, AT2G22500; and PIC2, AT5G14040) (Brautigam *et al.*, 2014; Jia *et al.*, 2015) was also observed in *C. gynandra*, but DIC expression was not enriched in BS cells of maize and *S. vidiris*. This suggests a requirement for the combination of the malate phosphate antiporter DIC with the phosphate/proton transporter PIC2 to pump malate into mitochondria in NAD-ME species, but not in NADP-ME species (Fig. 5).

### Global comparisons of the M and BS transcriptomes among monocot and dicot C_4_ plants

To define the extent to which gene expression patterns are conserved or divergent in monocot and dicot C_4_ plants, the same adjusted *P*≤0.05 between M and BS samples was applied as the definition of ‘differentially expressed genes’ for switchgrass, *S. viridis* (John *et al.*, 2014), maize (Chang *et al.*, 2012), and *C. gynandra* (Aubry *et al.*, 2014). Among *S. viridis*, maize, and *C. gynandra*, 5049 and 4631 transcripts, 6691 and 7647 transcripts, and 372 and 338 transcripts were enriched in M or BS cells, respectively. In our switchgrass transcriptome data set, 4630 and 5122 genes were considered as differentially expressed in M or BS cells, respectively (Supplementary Table S6). The higher numbers in switchgrass and maize are probably due to the highly duplicated and complex natures of their genomes (John *et al.*, 2014).

Switchgrass, *S. viridis*, and maize are close relatives among the C_4_ grasses (Fig. 1A). To determine if homologous genes underlie C_4_ photosynthetic development in C_4_ grasses, homology alignment was applied to differentially expressed genes in switchgrass, *S. viridis*, and maize (Fig. 5B). A total of 378 and 452 homologous genes are preferentially expressed in M and BS cells of these three C_4_ grasses. Pairwise comparison showed that *S. viridis* and maize share the most homologs that are preferentially expressed in M and BS cells, whereas fewer homologs are shared between *S. viridis* and switchgrass, and between switchgrass and maize. Classification into the same subtype contributes to this higher degree of similarity in M- and BS-enriched genes of maize and *S. viridis*, though switchgrass and *S. viridis* have a very close phylogenetic relationship (Fig. 1A) (Bennetzen *et al.*, 2012).

**Fig. 6.**
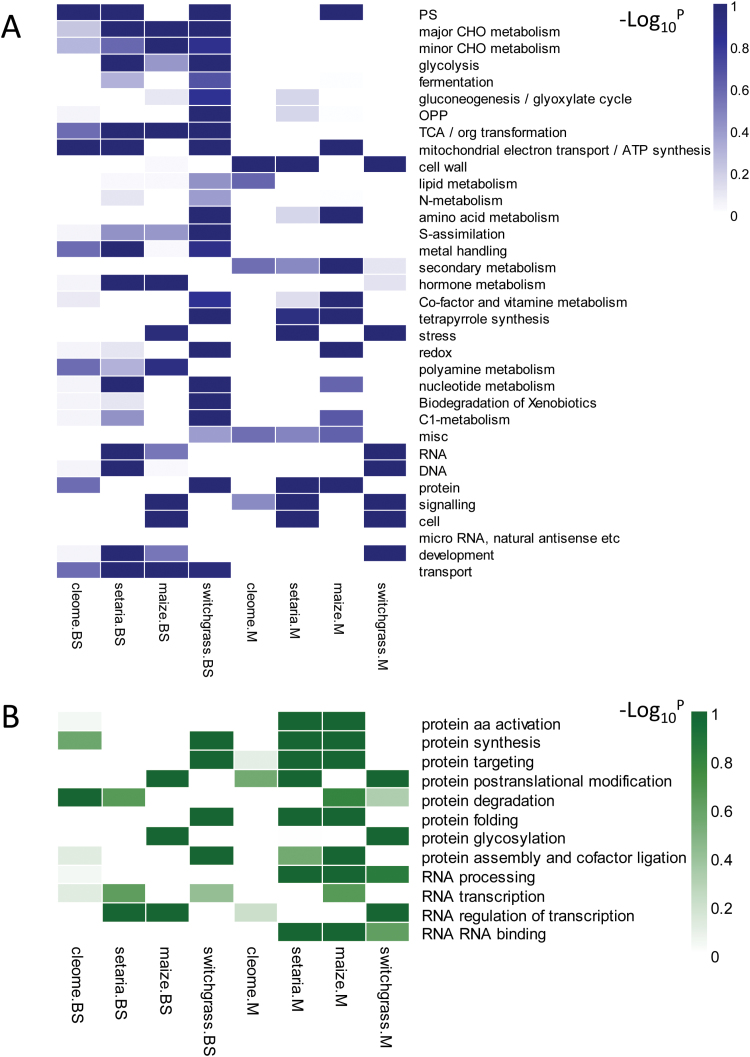
Functional distribution of BS and M cell type-enriched genes in switchgass, *S. viridis*, maize, and *C. gynandra*. Cell type-enriched functional groups in main categories (A) and in subcategories of protein and RNA (B) were identified by Fisher exact test (FDR<0.1 for each group). (This figure is available in colour at *JXB* online.)

To compare the functional differentiation between M and BS cells in C_4_ plants, the biological functions of cell-specific enriched genes were visualized using Mapman. Between 74% and 100% of the cell-specific enriched genes in the four C_4_ species could be assigned into 35 main categories (Fig. 6; Supplementary Table S7). To evaluate the convergence in the distribution of each category in C_4_ plants, we compared the gene number assigned into categories for pairs of species in M and BS cells, respectively. The Pearson’s correlation coefficient (*r* ^2^) ranged from 0.68 to 0.96, indicating a high degree of convergence in distribution of biological processes in the four distinct C_4_ species (Supplementary Table S7).

Using the Fisher exact test with FDR ≤0.1, we identified two and one, 12 and five, seven and nine, and 14 and seven main functional groups significantly enriched in M and BS cells of *C. gynandra*, *S. viridis*, maize, and switchgrass, respectively (Fig. 6A). Similarity in distribution of genes in certain categories was observed in these four C_4_ species. For example, considering that primary carbon assimilation is restricted to BS cells, transcripts in the categories of the TCA (tricarboxylic acid) cycle and major CHO (carbohydrate) metabolism were significantly enriched in BS cells of *S. viridis*, maize, and switchgrass, and moderately enriched in BS cells of *C. gynandra*. The preferential expression of genes involved in transport in BS cells is consistent with the fact that these cells are surrounded by vascular tissue for release and transport of metabolites to other plant parts (Christin *et al.*, 2009).

However, many differences were detected in functional enrichment between M and BS cells in the four C_4_ plants. For instance, transcripts encoding maize genes in the photosystem (PS) and mitochondrial electron transport/ATP synthesis categories were more abundant in M cells, whereas in the other three C_4_ species, these genes were preferentially expressed in BS cells. This is possibly because maize is depleted in PSII in the BS cells to lower oxygen production in these cells (Gowik and Westhoff, 2011).

Further analysis of subcategories within the main categories of protein and RNA revealed more divergence in functional enrichment within the four C_4_ plant species (Fig. 6B; Supplementary Fig. S7). Interestingly, genes involved in protein synthesis, targeting, and folding displayed preferential expression in BS cells in switchgrass and equal distribution in both cell types in *C. gynandra*, but strong enrichment in M cells in the two NADP-ME-type plants, *S. viridis* and maize. Strong enrichment in BS cells of genes involved in protein post-translational modification, protein glycosylation, and protein degradation mediated by AAA-type and ubiquitin proteases was observed in maize, whereas equal distribution in both cell types or moderate enrichment in M cells were found in switchgrass, *S. viridis*, and *C. gynandra*. In addition, genes involved in RNA regulation of transcripts were abundant in M cells of switchgrass, but are more abundant in BS cells of maize and *S. viridis*. This suggests that at both the post-transcriptional and translational levels, switchgrass, maize, and *S. viridis* may display different machineries for RNA transcriptional regulation and protein import, sorting, folding, assembly, and degradation that facilitate the preferential accumulation of proteins and accommodate protein homeostasis between M and BS cells. We suggest that the functional differentiation of post-transcriptional and translational regulatory mechanisms in M or BS cells of NAD-ME-type switchgrass, and NADP-ME-type maize and *S. viridis*, might be a result of their distinct evolutionary pathways, and/or may be associated with the accommodation of the different distribution of metabolites within M and BS cells of these two subtypes. For instance, NADP-ME-type plants require extra ATP produced by cyclic electron transport in BS cells to balance the production of NADPH for preventing photoinhibition or photodamage, and this is not required by NAD-ME-type plants (Nakamura *et al.*, 2013; Walker *et al.*, 2014).

### Convergence and divergence of cell type-enriched transcription factors in C_4_ plants

Given that differentially expressed TFs will play a major role in regulatory networks for the functional differentiation of M and BS cell types (Li *et al.*, 2010; Chang *et al.*, 2012), we annotated and identified TFs in the M and BS cell transcriptomes in switchgrass based on their homologs to Arabidopsis and rice in the Plant TF Database v3.0 (Jin *et al.*, 2014). Compared with the annotated TFs of *S. viridis* (John *et al.*, 2014), maize (Chang *et al.*, 2012), and *C. gynandra* (Aubry *et al.*, 2014), 243 and 175, 232 and 296, 274 and 412, and 23 and 20 TFs were identified as enriched genes in M and BS cells of switchgrass, *S. viridis*, maize, and *C. gynandra*, respectively (Supplementary Table S8). These genes were assigned into 60 TF families according to the Plant TF Database v3.0 (Jin *et al.*, 2014). Consistent with previous studies (Li *et al.*, 2010; Chang *et al.*, 2012), many TF families showed preferential cell type expression, indicating an overall differentiation in the regulatory networks underlying the functional differentiation of the two photosynthetic cell types in C_4_ leaves. However, similarities and differences were observed in the distributions of cell type preference of TF families within the four distinct C_4_ plants. Overall, maize and *S. viridis* shared more similarities in the distributions in TF families, resulting in Pearson’s correlation coefficients (*r* ^*2*^) of 0.70 and 0.89 in M and BS cells, respectively. In contrast, fewer similarities in distribution of cell type preference of TFs were shared between the monocot switchgrass and the dicot *C. gynandra*, although they belong to the same NAD-ME subtype.

To assess the potential co-option of TFs derived from a common ancestor, syntenic orthologs of TFs were determined between maize, *S. viridis*, and switchgrass. Switchgrass shared 34 and 13 TFs in M cells, and 44 and 18 TFs in BS cells as syntenic orthologs with *S. viridis* and maize, respectively. Among them, two and 10 syntenic ortholog TFs overlapped with M and BS cell-type enrichment, respectively, in the three C_4_ grass genomes. Interestingly, one and three syntenic ortholog TFs in maize with M and BS cell specificity, respectively, have been reported as regulatory candidates in differentiation of C_4_ Kranz anatomy (Wang *et al.*, 2013; Tausta *et al.*, 2014). For example, SCR is highly expressed in the BS in the three C_4_ grasses and has been proven to play an essential role in BS cell fate specification in plants (Wysocka-Diller *et al.*, 2000; Slewinski *et al.*, 2012; Cui *et al.*, 2014). This suggests that TFs might be in convergent evolution from a common ancestor and recruited in parallel in distinct C_4_ linages.

To examine further possible candidates in BS establishment, homologous TFs with BS cell enrichment in four C_4_ plants were identified, and MYB59, which is reported to regulate the cell division process in Arabidopsis roots (Cominelli and Tonelli, 2009; Mu *et al.*, 2009), was found to be significantly enriched in BS cells of all four C_4_ species. MYB59 in maize was listed as a candidate in regulating C_4_ Kranz formation in a previous study (Wang *et al.*, 2013; Tausta *et al.*, 2014). It is possible that MYB59 may play a role in controlling BS cell size in C_4_ species. In addition, transcriptional activators of lignification such as MYB42 (Hussey *et al.*, 2013) were more abundantly expressed in the BS cells in the three monocot C_4_ grasses than in *C. gynandra*, and the transcriptional repressor of lignification MYB4 (Shen *et al.*, 2012) was highly expressed in M cells in switchgrass. This is consistent with the thicker cell walls in the BS cells of monocot as compared with dicot plants (Majeran *et al.*, 2005), and suggests that the C_4_ grasses might share similar regulatory mechanisms for secondary cell wall formation in BS cells.

### Conclusions

The differentiation of M and BS cells with their specific accumulation of transcripts in C_4_ leaves is considered a hallmark of the C_4_ pathway (Burgess and Hibberd, 2015). Enzymatic and mechanical isolation or laser capture micro-dissection of M and BS cells have been applied in quantitative transcriptomics of specific cell types (Li *et al.*, 2010; Chang *et al.*, 2012; Aubry *et al.*, 2014; John *et al.*, 2014). Here we employed manual dissection of switchgrass M and BS cells, which is a simple and inexpensive means of obtaining a small specimen for further cell type-specific transcriptome analysis (Outlaw and Zhang, 2001). This has enabled, to the best of our knowledge, the first estimate of mRNA profiles of M and BS cells in a monocot NAD-ME subtype C_4_ plant.

A high degree of convergence in the distribution of C_4_ marker genes in M/BS cells was observed in the four distinct C_4_ species; monocot NAD-ME-type switchgrass, monocot NADP-ME-type *S. viridis* and maize, and dicot NAD-ME-type *C. gynandra* (Fig. 4A). Furthermore, these species shared a high degree of convergence in distribution of gene functional categories in M/BS cells based on analysis of the Pearson’s correlation coefficient (*r* ^2^) (Fig. 6; Supplementary Table S7). Genes grouped into the categories of TCA and major CHO metabolism were significantly or moderately enriched in BS cells in all four C_4_ species. A similar preferential expression of SCR and MYB59 TFs was found in C_4_ grasses and in all four C_4_ species, respectively (Supplementary Table S8). We conclude that the transcript abundance of core components in C_4_ photosynthesis, the distribution of gene function, and the mechanism of establishment of BS might be convergent in these four independent lineages of C_4_ plants.

The pathways of C_4_ photosynthesis are divided into three basic biochemical subtypes based on the major decarboxylating enzymes to release CO_2_: NAD-ME, NADP-ME, and PEPCK (Gowik and Westhoff, 2011). However, the differentiation observed in our comparative transcriptomes of NAD-ME and NADP-ME subtypes goes beyond the decarboxylating enzymes. Besides aminotransferase (Fig. 4A), the NAD-ME type differs from the NADP-ME type by the differential compartmentation of specific metabolite transporters, such as sodium:pyruvate and proton:pyruvate co-transporters (Supplementary Table S3), and by more general cell-specific functional enrichment, such as RNA regulation and protein biogenesis/homeostasis (Fig. 6). The abundance of transcripts functioning in protein synthesis, folding, and assembly is more prominent in BS and M cells of switchgrass and *C. gynandra* than in *S. viridis* and maize. C_4_ plants might have undergone convergent processes such as gene duplication, anatomical pre-conditioning, and compartmentalization of C_4_ core enzymes to evolve C_4_ photosynthesis (Sage, 2004), but may have undergone divergent processes for the optimization of M and BS cell co-ordination that are not directly associated with the C_4_ pathway.

## Supplementary data

Supplementary data are available at *JXB* online.

Figure S1. Cross- and longitudinal sections of a switchgrass leaf.

Figure S2. Qualitative and quantitative analysis of total RNA from manually isolated BS and M cells using a Bioanalyzer.

Figure S3. Validation of C_4_-related gene expression in switchgrass by qRT–PCR.

Figure S4. Phylogenetic comparison of switchgrass NAD-ME with NAD-ME protein sequences in other species.

Figure S5. Phylogenetic comparison of switchgrass NADP-ME with NADP-ME protein sequences in other species.

Figure S6. Analysis of syntenic genes of NAD-ME and NADP-ME in grasses

Figure S7. Cell type-enriched functional groups in subcategories of protein degradation identified by Fisher exact test.

Table S1. Raw and normalized data.

Table S2. The fold change of C_4_ enzyme activity in age- and area-related experiments in switchgrass leaves.

Table S3. List of C_4_-related genes in the switchgrass M and BS transcriptomes.

Table S4. Sequences of primers used in the present work.

Table S5. Convergence in abundance of C_4_ marker genes in switchgass, *S. viridis*, maize, and *C. gynandra*.

Table S6. List of differentially expressed genes in M and BS cells from switchgrass, *S. viridis*, maize, and *C. gynandra*.

Table S7. Functional distribution of differentially expressed genes in M and BS cells from switchgass, *S. viridis*, maize, and *C. gynandra*.

Table S8. List of differentially expressed TFs in M and BS cells from switchgrass, *S. viridis*, maize, and *C. gynandra*.

Supplementary Data
