# Supplementary material for: Comparative cell-specific transcriptomics reveals differentiation of C4 photosynthesis pathways in switchgrass and other C4 lineages
Source: J Exp Bot. 2016 Feb 19;67(6):1649–62. doi: 10.1093/jxb/erv553 (PMC4783356; doi:10.1093/jxb/erv553)
Supplement: Supplementary Data [file supp_67_6_1649__index.html]

Comparative cell-specific transcriptomics reveals differentiation of C4 photosynthesis pathways in switchgrass and other C4 lineages — Comparative cell-specific transcriptomics reveals differentiation of C4 photosynthesis pathways in switchgrass and other C4 lineages — Supplementary Data 

# Comparative cell-specific transcriptomics reveals differentiation of C4 photosynthesis pathways in switchgrass and other C4 lineages

## Supplementary Data

Data files

- supplementary\_figures\_S1\_S7.pdf - Supplementary Data
- supplementary\_data\_files\_S1.xlsx - Supplementary Data
- supplementary\_data\_files\_S2.xlsx - Supplementary Data
- supplementary\_data\_files\_S3.xlsx - Supplementary Data
- supplementary\_data\_files\_S4.xlsx - Supplementary Data
- supplementary\_data\_files\_S5.xlsx - Supplementary Data
- supplementary\_data\_files\_S6.xlsx - Supplementary Data
- supplementary\_data\_files\_S7.xlsx - Supplementary Data
- supplementary\_data\_files\_S8.xlsx - Supplementary Data
