# Supplementary material for: Comparative cell-specific transcriptomics reveals differentiation of C4 photosynthesis pathways in switchgrass and other C4 lineages
Source: J Exp Bot. 2016 Feb 19;67(6):1649–62. doi: 10.1093/jxb/erv553 (PMC4783356; doi:10.1093/jxb/erv553)
Supplement: Supplementary Data [file supp_erv553_supplementary_figures_S1_S7.pdf]

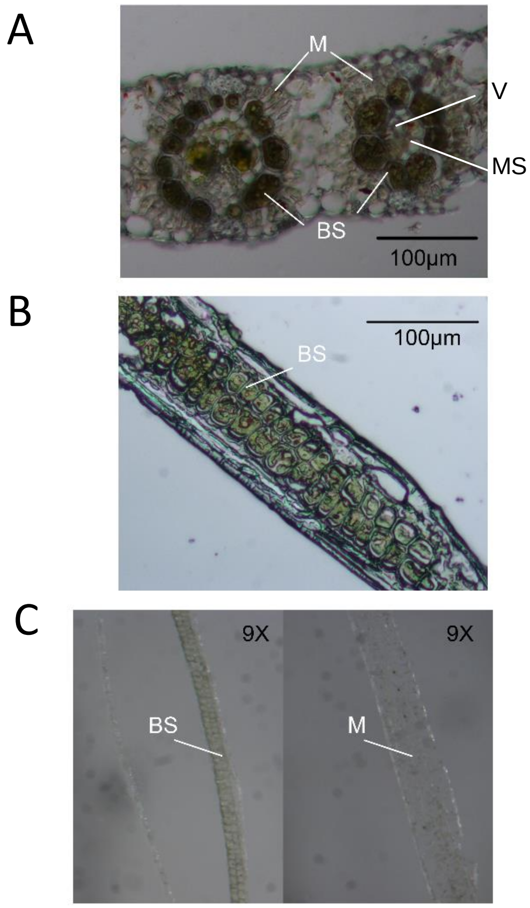

Figure S1. Cross and longitudinal sections of switchgrass leaf.
(A) Cross-sections of switchgrass leaf blades with 50 µm thickness; (B) Longitudinal-sections of switchgrass leaf blades with 10µm thickness; (C) Longitudinal cryosections, with 10 µm thickness, of bundle sheath and mesophyll cells under the fluorescence stereo microscope, respectively. M, mesophyll cells; BS, bundle sheath cells; MS, mestome sheath; V, vascular tissue.

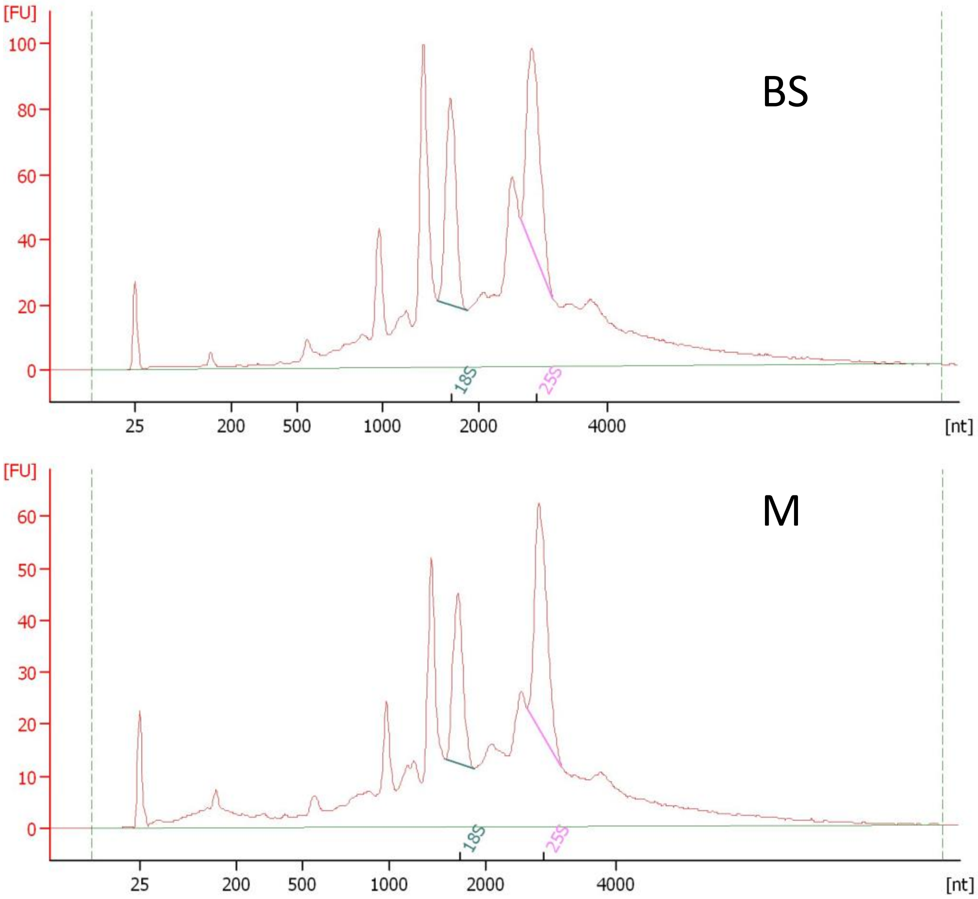

Figure S2. Qualitative and quantitative analysis of total RNA from manually isolated BS and M cells using a Bioanalyzer.

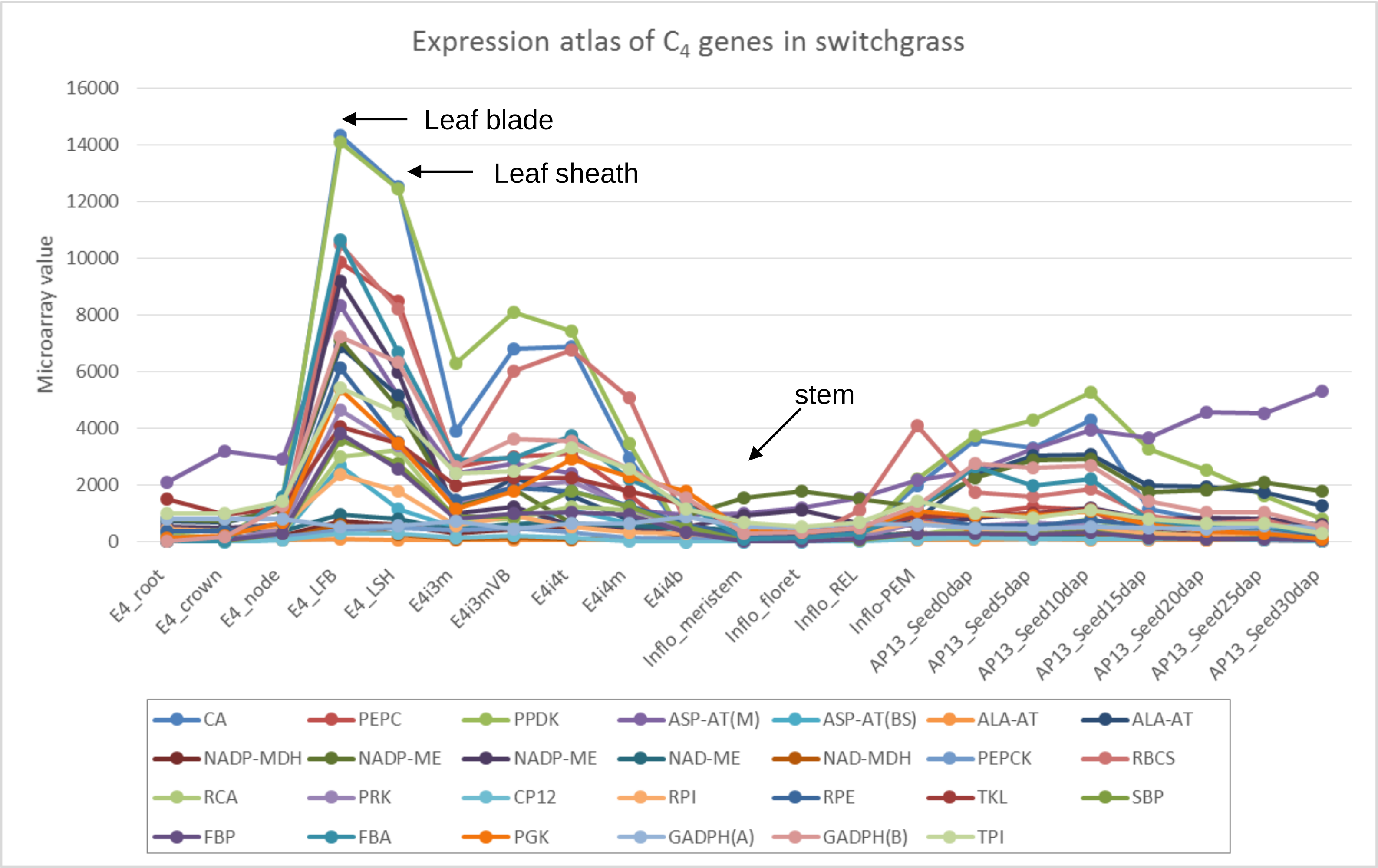

Figure S3. Validation of C_4-related gene expression in switchgrass by Q-PCR.
(A) Expression atlas of C_4 genes in switchgrass, showing transcript levels in 21 major tissues and organs from the Switchgrass Functional Genomics Server (http://switchgrassgenomics.noble.org/). The corresponding microarray probes are provided in Supplemental Data File 4.

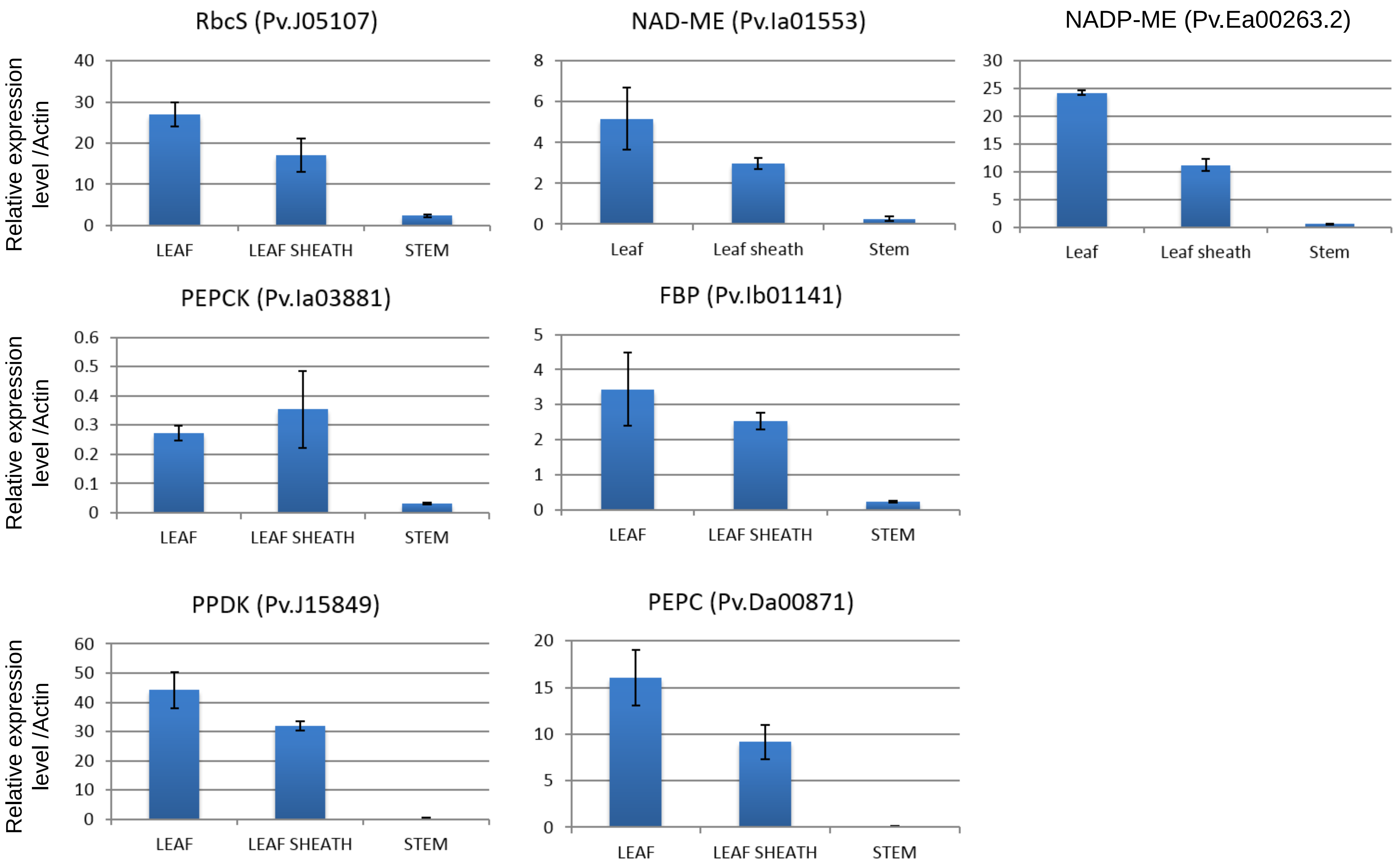

Figure S3. Validation of C4-related gene expression in switchgrass by Q-PCR.
(B) qRT-PCR was performed to verify the expression patterns of RbcS, NAD-ME, NADP-ME, PEPCK, FBP, PPDK and PEPC in leaf, leaf sheath and stems. Expression values were normalized to expression levels of actin. The results are the means of triplicate measurements of three biological replicates. Error bars represent SD of three biological replicates.

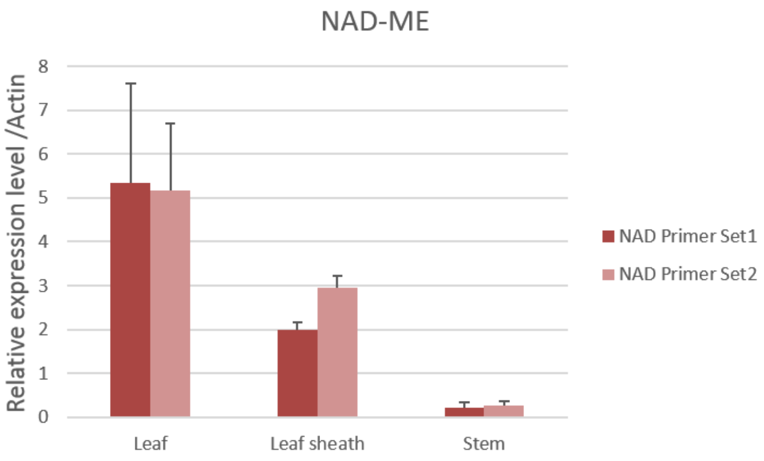

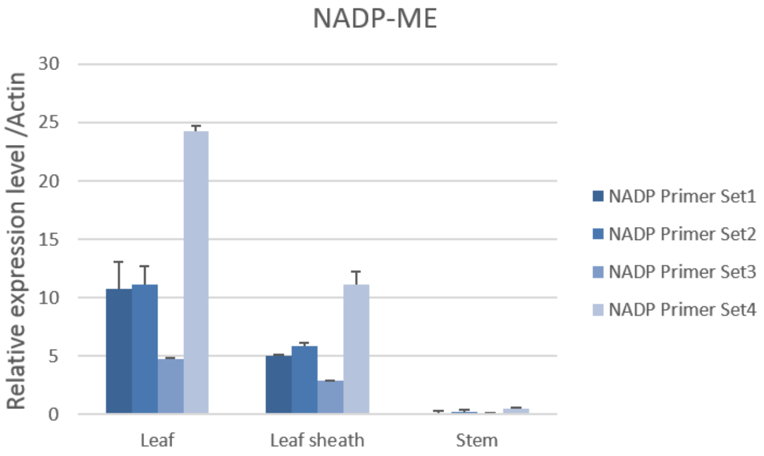

Figure S3. Validation of C4-related gene expression in switchgrass by Q-PCR.
(C) qRT-PCR was performed to verify the expression patterns of NAD-ME and NADP-ME using multiple primer sets in leaf, leaf sheath and stems. Expression values were normalized to expression levels of actin. The results are the means of triplicate measurements of three biological replicates and error bars represent SD of three biological replicates.

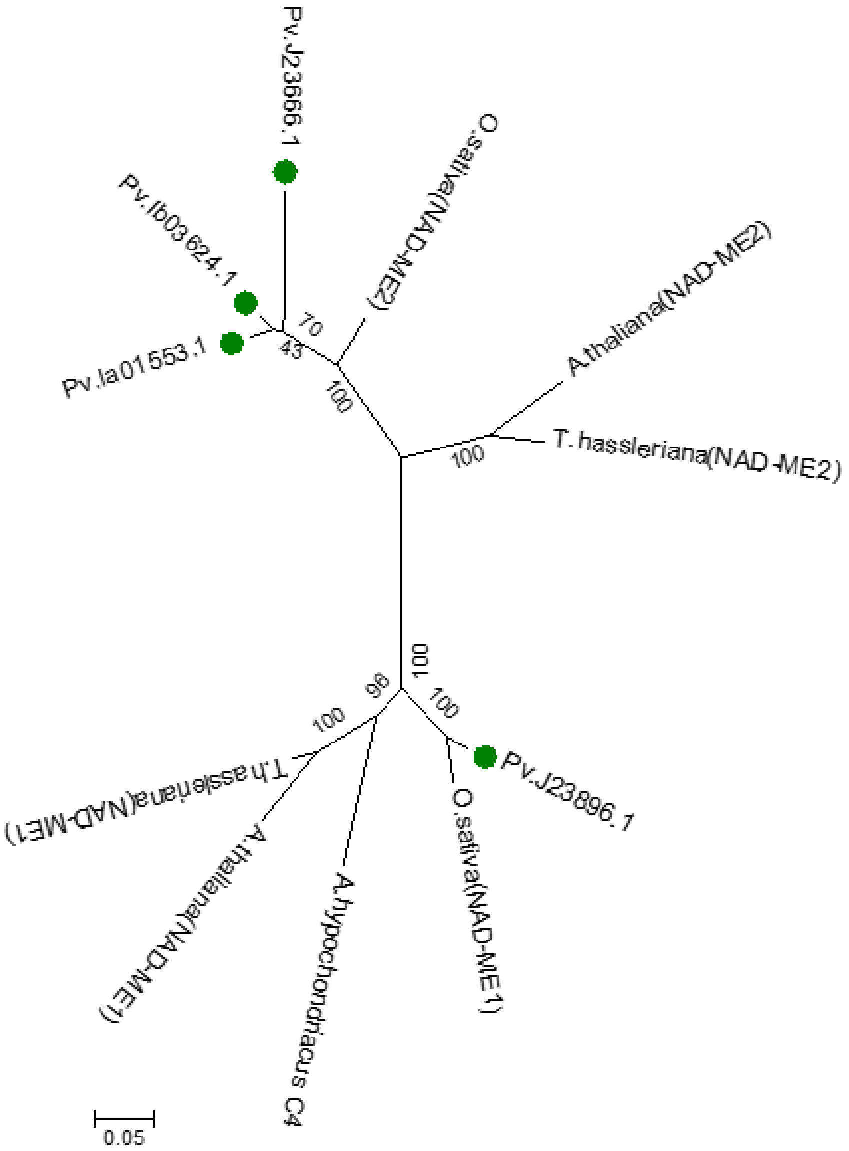

Figure S4. Phylogenetic comparison of switchgrass NAD-ME with other NAD-ME protein sequences. The NAD-MEs from switchgrass are marked with green spots. Phylogenetic analysis was conducted by Mega 6 program. Numbers indicate the percent value of 1,000 bootstrap replications.

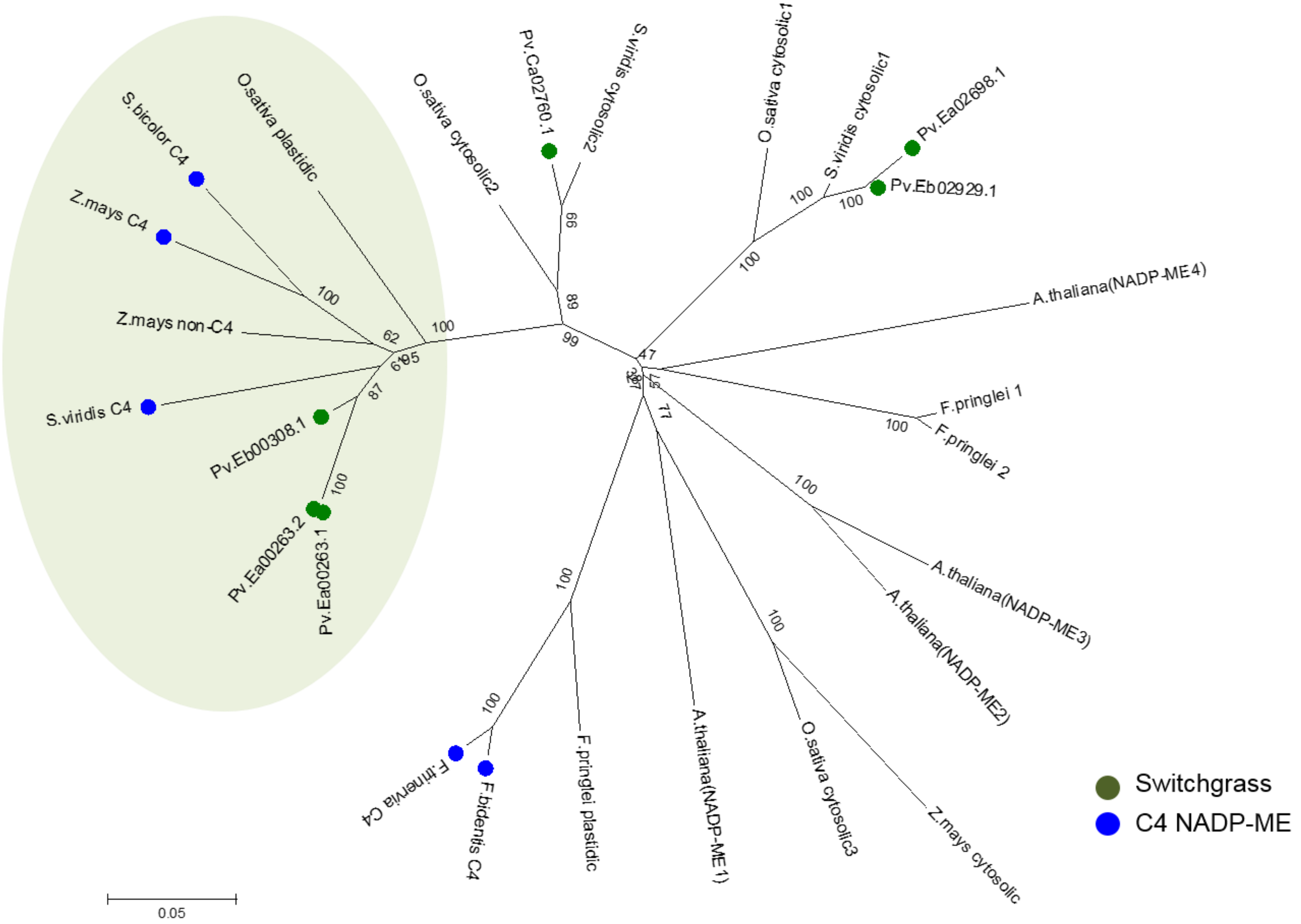

Figure S5. Phylogenetic comparison of switchgrass NADP-ME with other NADP-ME protein sequences. The NADP-MEs from switchgrass are marked as green spots and the NADP-MEs functioning in C4 photosynthesis in other species are marked as blue spots. Phylogenetic analysis was conducted by Mega 6 program. Numbers indicate the percent value of 1,000 bootstrap replications.

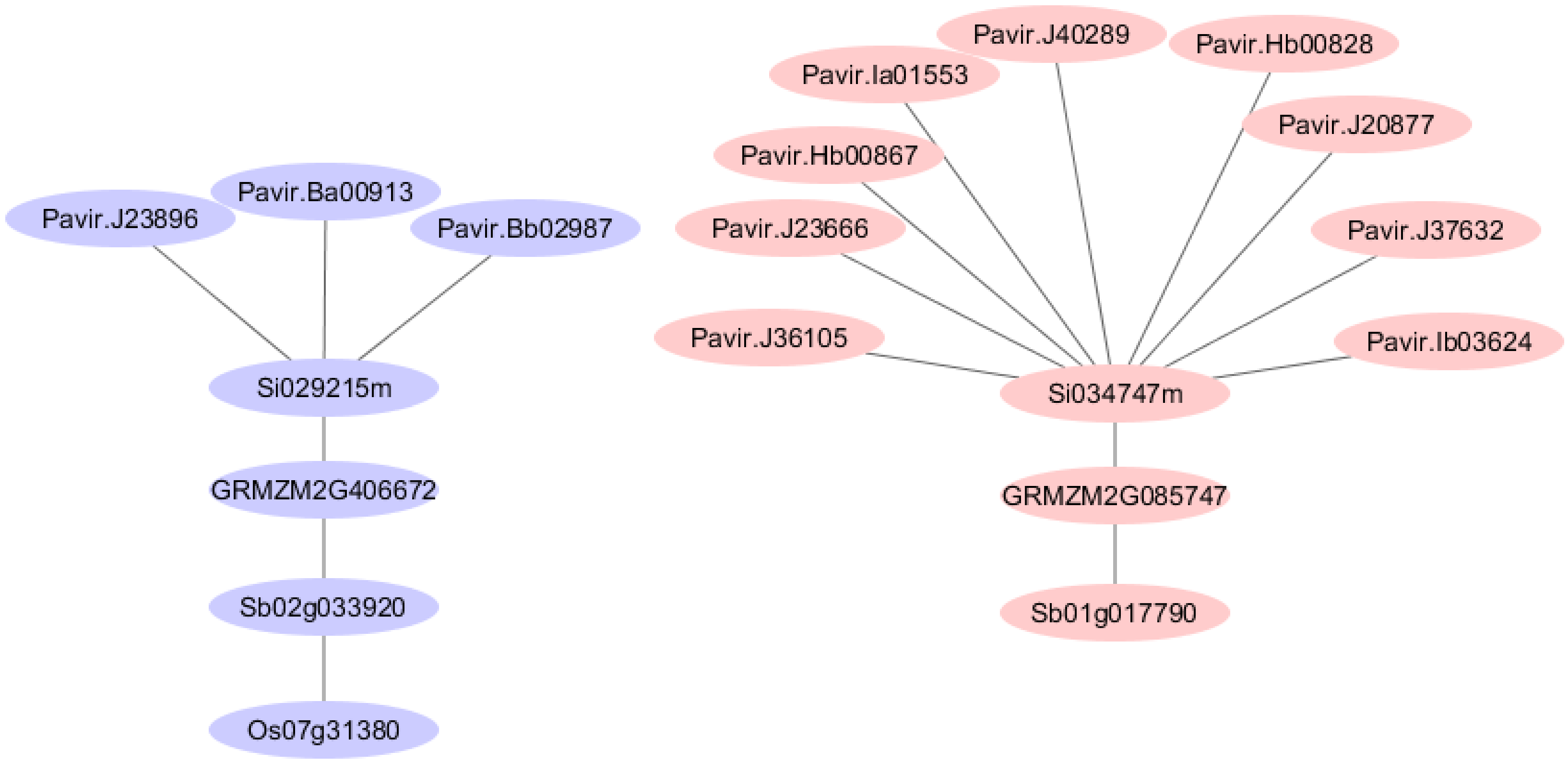

Figure S6. Analysis of syntenic genes of NAD-ME and NADP-ME in the grasses.
(A) Syntenic orthologs of NAD-ME in the grasses. Blue color represents NAD-ME type 1 and red color represents NAD-ME type 2.

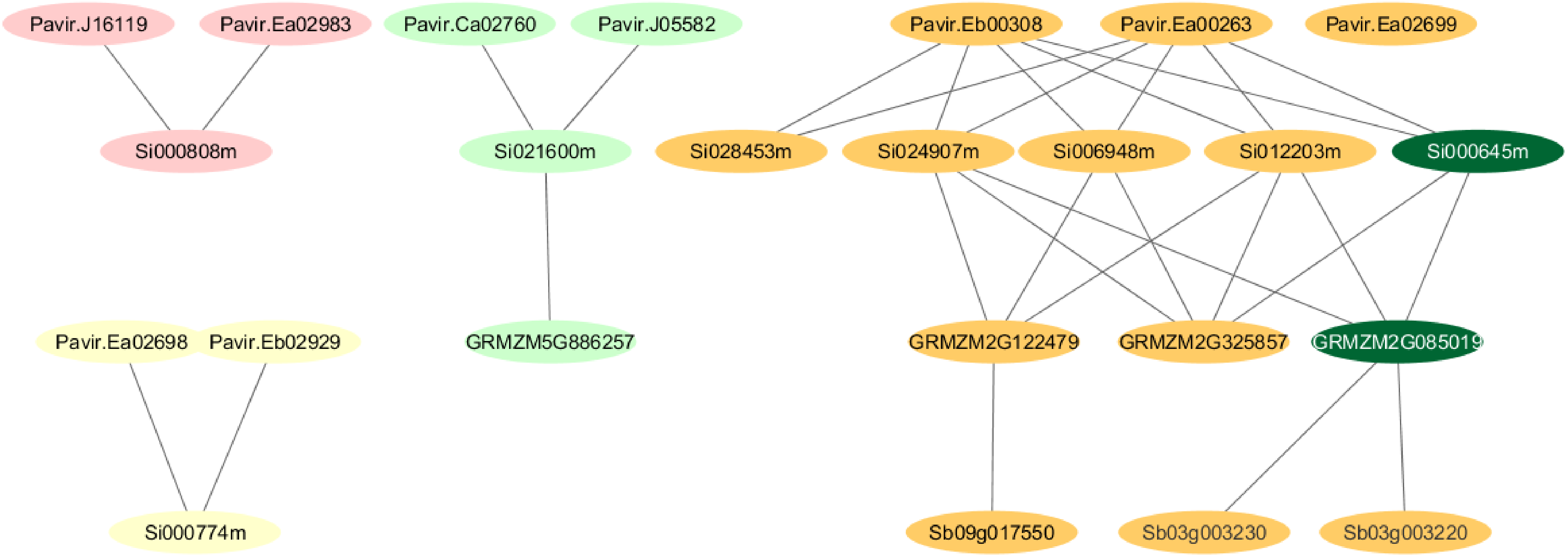

Figure S6. Analysis of syntenic genes of NAD-ME and NADP-ME in the grasses.
(B) Syntenic orthologs of NADP-ME in the grasses. Yellow, cytosolic NADP-ME 1; light green, cytosolic NADP-ME 2; Red, cytosolic NADP-ME 3; orange, non-C4 NADP-ME and dark green, C4 NADP-ME.

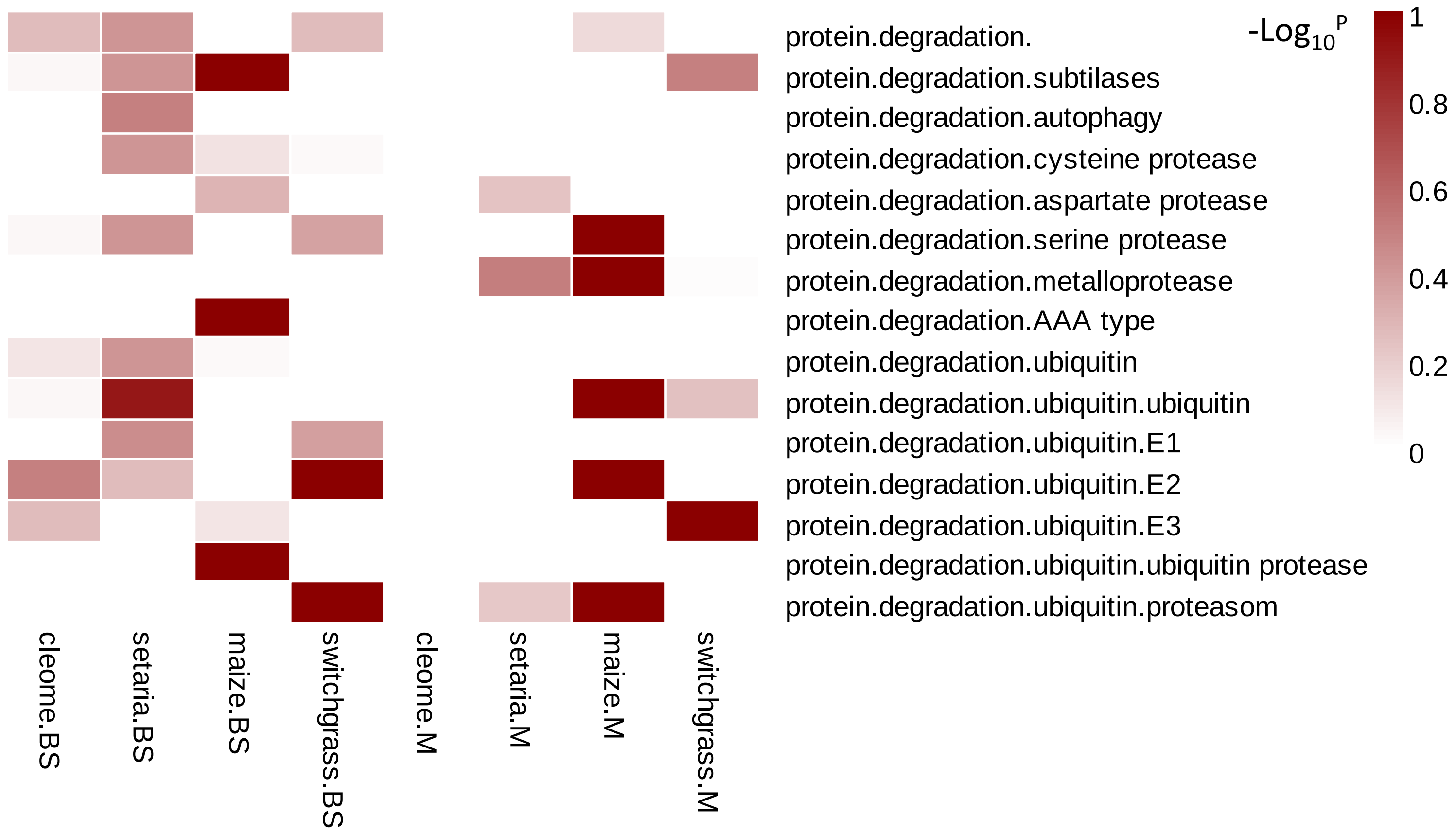

Figure S7. Cell type-enriched functional groups in sub categories of protein degradation were identified by Fisher exact test (FDR<0.1 for each group).
